# The Impacts of Microgravity on Bacterial Metabolism

**DOI:** 10.3390/life12060774

**Published:** 2022-05-24

**Authors:** Gayatri Sharma, Patrick D. Curtis

**Affiliations:** Department of Biology, University of Mississippi, University, MS 38677, USA; gsharma@go.olemiss.edu

**Keywords:** primary metabolism, secondary metabolism, spaceflight, radiation, microgravity

## Abstract

The inside of a space-faring vehicle provides a set of conditions unlike anything experienced by bacteria on Earth. The low-shear, diffusion-limited microenvironment with accompanying high levels of ionizing radiation create high stress in bacterial cells, and results in many physiological adaptations. This review gives an overview of the effect spaceflight in general, and real or simulated microgravity in particular, has on primary and secondary metabolism. Some broad trends in primary metabolic responses can be identified. These include increases in carbohydrate metabolism, changes in carbon substrate utilization range, and changes in amino acid metabolism that reflect increased oxidative stress. However, another important trend is that there is no universal bacterial response to microgravity, as different bacteria often have contradictory responses to the same stress. This is exemplified in many of the observed secondary metabolite responses where secondary metabolites may have increased, decreased, or unchanged production in microgravity. Different secondary metabolites in the same organism can even show drastically different production responses. Microgravity can also impact the production profile and localization of secondary metabolites. The inconsistency of bacterial responses to real or simulated microgravity underscores the importance of further research in this area to better understand how microbes can impact the people and systems aboard spacecraft.

## 1. Introduction

One of the many interesting aspects of bacteria is their ability to successfully withstand and colonize virtually any niche in spite of growth-limiting challenges, hostile surrounding conditions, and even bactericidal measures [1,2]. They are ubiquitous, present in nearly every environment from the abyssal zone to the stratosphere at heights up to 60 km, from arctic ice to boiling volcanoes [3,4,5,6]. They can also be found in space-faring vehicles, which, with their low gravity and high radiation conditions, provide a set of stresses unlike on Earth. With upcoming long-term space explorations, it is becoming increasingly important to understand the microbial responses to these stresses and how they may affect the function of space-faring vehicles and the occupants within them [7]. Significant progress has been made towards the understanding of the effects of space environmental factors, both real and simulated, over the past 60 years [8]. Multiple studies have shown that bacterial cells demonstrate altered physiological characteristics, including proliferation rate, cell division, virulence, biofilm formation, motility, susceptibility to antibiotics, and cellular metabolism [7,9]. Metabolism can be defined as the sum of all reactions in a living cell aimed at maintenance, development, and division. Bacterial metabolism is comprised of primary metabolites, the intracellular molecules that enable growth and proliferation [10,11,12] and secondary metabolites, predominantly extracellular molecules that facilitate a microbe’s interaction and adaptation with its environment [10,12]. Primary metabolites can include amino acids, nucleotides, and fermentation end products such as ethanol and organic acids [11,12]. They are found intracellularly and are usually charged to prevent diffusion through the cell membrane [10,13]. On the other hand, secondary metabolites are predominantly low molecular weight compounds, extracellular, and are typically uncharged and non-polar so that they can pass through the cell membrane [10]. They are usually produced in late-exponential and stationary phases [10], and are not directly associated with growth, development, or division of bacteria. These specialized products are most notable for their use in healthcare settings as antimicrobial, antiparasitic, and antitumor agents [11,14,15] Though these metabolisms are sometimes thought to be fundamentally separate, they are intimately linked. Many of the intermediates in primary metabolism are precursors of secondary metabolites, and cells have evolved complex molecular switches linking primary and secondary metabolic pathways. These include high expression of the secondary metabolism genes at specific times in the cell cycle and controlling the flow of primary metabolites (carbon and nitrogen) through different pathways by feedback regulation [10,11,15,16].

Many factors can influence a bacterial metabolism, which in turn can affect the physiological properties of an organism. A potent factor is cellular stress. Spaceflight is unfriendly frontier for organisms of all sizes, including bacteria. This review will discuss the stressful conditions bacterial cells experience under both spaceflight and simulated microgravity condition and the known primary and secondary metabolic changes that occur as a result of those stressful conditions. Integrated information of primary and secondary pathways could contribute to pivotal information regarding the molecular basis of diverse responses demonstrated by bacteria to adapt to the harsh conditions of spaceflight.

## 2. Stresses Imposed by Spaceflight

The extraterrestrial environment, which is characterized by a high vacuum, intense radiation and low magnitude of gravity, provides stressful conditions to any form of life [8,17]. Microgravity (10^−3^ to 10^−6^ g), which is the gravity range seen on the International Space Station, has been shown to impact organisms of all sizes [7,8,17,18,19]. Some observed changes to microgravity include global alterations in gene expression [20,21,22] 3-D aggregation of bacterial cells into tissue-like architecture [18,23] and changes in bone density of humans [18,19] and developmental patterns of plants [24]. Bacteria, due to their extremely small mass, do not experience the same level of gravitational force on Earth as compared to macroscopic organisms, so it would be easy to assume that bacteria would not become overly stressed by microgravity. However, adaptive transcriptional and metabolic alterations have been observed in bacteria in spaceflight conditions relative to their Earth behavior [7,8]. All microorganisms in space-faring vehicles, originating through contaminants from Earth, components of experiments, or as the normal microbiota of crew members [25,26,27,28], experience the stressful effects generated from altered gravity and cosmic radiations. Experiments examining the effect of microgravity on bacterial physiology have been conducted two different ways: (a) spaceflight experiments on actual spacecraft such as the International Space Station (ISS), and (b) in Ground-Based Facilities (GBFs) that mimic microgravity using Clinostats and Rotating Wall Vessels [29]. Actual spaceflight experiments are the gold-standard in biological microgravity research, but the high cost of spaceflight and limited availability of spaceflight opportunities often necessitate simulation in GBFs. Forces, such as centrifugal forces, sedimentation of a particle (as a function of its mass and the viscosity of the medium) [30], phase shifts of mobile particles as a consequence of rotation, friction experienced by a mass as a result of rotation, are not experienced by cell under real microgravity. It is impossible to abolish the 1*g* force of gravity on Earth. However, randomizing the direction of gravity by constant rotation of a sample causes cell to essentially experience constant “free fall”, thereby somewhat mimicking the microgravity environment [31]. The rate of rotation should be maintained where there is a balance between centrifugal and centripetal forces, and horizontal axis of rotation to achieve nullification of the force of gravity [30,32,33,34].

The changes in physiological responses obtained using GBFs are frequently found to be similar to those observed in comparable spaceflight experiments [33]. However, the constant free-fall experienced by cells in GBFs is not exactly the same condition experienced by cells in true spaceflight microgravity, and cells in GBFs do not experience any of the other condition changes of spaceflight, such as increased exposure to radiation. It cannot be assumed that both experimental designs always induce similar responses.

It is not clear how bacteria can sense a change in gravity, as no gravity sensing mechanism has been identified in bacteria. It is possible that bacteria contain mechano-transduction systems that can sense and respond to low-shear stress and changes in gravitation forces [35,36]. Aside from possible mechano-sensing systems, it is hypothesized that the observed bacterial physiological changes during spaceflight are due to microgravity altering the extracellular environment of cells [35]. In microgravity, bacterial cells experience lower than normal levels of shear stress, low turbulence, and a relative lack of sedimentation as compared to normal gravity conditions [35,36]. The lack of gravity-driven forces and flows (namely buoyancy, sedimentation, and convection) cause the movement of molecules to and from the cell to become limited by diffusion [8,36,37]. This means the movement of nutrients to cells and waste products away from cells is limited to Brownian motion [38]. The reduction of extracellular nutrient availability and the accumulation of bacterial byproducts near the cell will have dramatic consequences for the organism, particularly in cellular metabolism [8,38,39]. It is perhaps unsurprising that different responses to microgravity have been observed between non-motile and motile cells, the latter having the ability to escape their local microenvironment [32,37,40,41,42,43,44,45]. However, studies have shown that non-motile bacteria or motile strains with impacted motility show a shortened lag phase and an increase in cell density under spaceflight microgravity conditions [35,37,38,42,44,46]. Given that non-motile cells will quickly consume the local nutrients of a diffusion limited environment, that inhibitory by-products will build up in the local extracellular environment more quickly, and the lack of motility will not allow cells to escape this condition, it seems counterintuitive that non-motile cells would demonstrate greater proliferation under microgravity. There are different theories to explain this phenomenon. One hypothesis suggests this is a direct effect of reduced gravity causing small changes in the cellular machinery or the cell membrane which alters the cell’s energy requirements and stimulates growth [47]. Another hypothesis suggests the increased cell proliferation is due to an indirect effect of the lack of sedimentation [35]. The lack of sedimentation in microgravity allows bacteria to remain near their by-products in the quiescent environment. Some of these by-products are enzymes or cofactors that may be beneficial to growth. Since bacteria under low shear conditions get constant exposure of such by-products, they come out of lag phase sooner than ground controls. In contrast, the cells on Earth sediment away from these beneficial byproducts, and as a result require more time to come out of lag phase [35]. Therefore non-motile cells proliferate faster due to an early and elongated exponential phase as suggested in both spaceflight [32,37,38,40,41,42,43,44,45,48] and simulated microgravity based studies [49].

Other extreme conditions of spaceflight include extreme heat and cold cycling, elevated CO_2_ levels, and high energy radiation. Radiation in particular is a potent stress. Space vehicles can experience Galactic Cosmic Radiation (GCR), Solar Cosmic Radiation (SCR), and a radiation belt trapped by the Earth’s magnetosphere [8,17] These are all ionizing radiation and are composed of high-energy particles; GCR is composed of high-energy protons (90%), α-particles (9%), and heavy particles (1%), and SCR is composed of protons and electrons, α-particles, and heavy particles [17,50,51]. Experiments conducted to study the effect of UV- radiation have observed greater incidence of mutations, and generation of greater oxidative stress [37]. Moreover, it has always been suggested by many terrestrial and spaceflight studies that there is a variation in the number of genes expressed depending on the exposure to different types of radiations including UVA, UVB, and UVC [37,50,51,52]. Although these stresses could have a collaborative effect on bacterial metabolism in space, determining individual impacts of such stressors is outside the scope of this review and all such effects can be attributed solely to microgravity.

## 3. Metabolic Changes under Spaceflight and Simulated Microgravity Conditions

The metabolic changes exhibited by microbes in response to the extreme environments of spaceflight have attracted increasing attention. Studies over the years have shown that microorganisms survive in space vehicles by exhibiting changes in expression at the transcriptional and translational levels along with alterations in metabolic pathways [7,8,29]. Initially, studies were mainly focused on understanding the impact of spaceflight on specific aspects of microbial gene expression, physiology or pathogenesis. However, several transcriptomics and proteomics approaches have recently opened a window for a deep insight into the molecular responses of microgravity-grown bacteria, revealing changes in global expression, metabolic function, and regulation of the genes or proteins in space grown microbes [7,17,36].

### 3.1. Primary Metabolism

Primary metabolism studies over the past century have provided a quantitative, detailed and holistic picture of many organisms under terrestrial conditions [10,53,54]. This kind of understanding is not yet available under spaceflight conditions, which is a growing hub for microbial communities. Metabolic studies under microgravity thus far have suggested the broad trend of overexpression of genes associated with starvation and enhanced trans-membrane influx, indicative of nutrient depletion [22,29,35,36]. Under terrestrial conditions starvation can lead cells either to undergo growth arrest or manipulate their metabolism to harvest other available energy. Cells either feed on internal resources or devote more of their limited resources to the transcriptional changes needed to broaden the search for alternative sources of carbon [55]. Under both situations, different metabolic pathways are activated to increase their ability to rapidly switch carbon catabolic pathways if a new substrate becomes available [56]. Microgravity exacerbates the starvation condition due to nutrient diffusion limitation [35]. Any changes at a gene level or metabolite level can have a possible implication on overall bacterial metabolism including glucose catabolism, amino acid metabolism, and lipid metabolism.

One general trend noted among several studies is upregulation of carbohydrate metabolism genes, which is thought to be a by-product of nutrient limitation, which leads to an increase in metabolic rate. A study on *Stenotrophomonas maltophilia* under simulated microgravity displayed an increased growth rate which was suggested to be related to the upregulation of genes involved in catalysis of carbohydrates and amino acids, along with energy metabolism and secondary metabolite biosynthesis and transport [57]. These metabolic changes are consistent with reduced extracellular nutrient transport [35]. A study of *E. coli* grown on the ISS reported the activation of 69% of glucose catabolism genes along with 74% of the genes associated with metabolism that are not directly involved in glucose. These data indicate a global impact on metabolic activity in *E. coli*, suggesting an overall increase in metabolic rate [35]. As one example, the study reported overexpression of the *thiEFGHS* operon, which codes for thiamine biosynthesis [35]. The overexpression of these genes indicates an increased synthesis of thiamine, which is an important cofactor needed for carbohydrate metabolism [58]. In terrestrial conditions, it has been reported that *E. coli* accumulates adenosine thiamine triphosphate (a form of thiamine) when energy substrates or carbon sources are lacking [59]. Thus, the observed increased thiamine synthesis under microgravity implies starvation conditions, possibly due to reduction of extracellular nutrient availability. The *E. coli* microgravity study also reported increased expression of 88% of genes responsible for glucose catalysis into organic acids, predominantly acetate [35]. Glucose can be metabolized into acetate via different pathways including: (a) pyruvate dehydrogenase complex (PDHc), (b) pyruvate-formate lyase (PflB), and (c) pyruvate oxidase (PoxB). Of these three mechanisms, only the *poxB* gene showed an increased expression in microgravity. This enzyme is known to be expressed in late-exponential and stationary phase *E. coli* cultures [60], and is indicative of microaerobic conditions [61]. The higher cell density observed under spaceflight, which already in itself is limited in diffusion of oxygen, could have further decreased the concentration of dissolved oxygen in the medium resulting in overexpression of PoxB and metabolism of glucose directly into acetate. Incidentally, PoxB is known to bind thiamin pyrophosphate [62].

Interestingly, discrepancies were observed in expression of genes for alternative carbon source utilization. An *E. coli* study on the ISS showed overexpression of *malE* and *lamB* genes, which are essential for the transport of maltose into the cell, even though the experiment used glucose as the sole carbon source, suggesting the cells broadened the search for alternative sources of carbon even if they were unavailable [35]. Contrary to the previous results, simulated microgravity grown of *Stenotrophomonas maltophilia* showed weak utilization of several carbon sources as compared to controls, including D-arabitol, myo-inositol, D-aspartic acid, L-pyroglutamic acid, quinic acid, and D-lactic acid methyl ester, suggested the inhibition of certain metabolic pathways under simulated microgravity condition [57]. It is not clear if the different response are due to the different bacteria or the difference between actual spaceflight and simulated microgravity.

Proteomics analysis of *E. coli* aboard the Shenzhou VIII spacecraft reported the downregulation of genes associated with arginine and proline catabolism via transcriptomic and proteomic analyses [13,63]. Similarly, proteomic analysis of *P. aeruginosa* under spaceflight conditions showed down-regulation of ArcA, an enzyme associated with the fermentation of arginine [64]. A study on *B. subtilis* reported that genes of arginine biosynthesis were up-regulated during spaceflight aboard the ISS [65]. These results indicate that the regulation of arginine and proline metabolism is particularly important for spaceflight. The reason likely involves oxidative stress, which can be a potent stress during spaceflight due to high ionizing radiation. Proline has many functions in the cell; aside from being a structural amino acid and a carbon and nitrogen source, proline acts as an organic osmolyte providing cellular protection against abiotic stresses such as drought and osmotic shock. Additionally, catabolism of proline generates electrons that can enter the electron transport chain, but also produces reactive oxygen species that can have toxic effects on the cell. Therefore, while the oxidative catabolism of proline can provide carbon and nitrogen, downregulation of proline catabolism could lead to increased intracellular proline levels, which would help prevent osmotic stress, while also avoiding generating undue oxygen stress, both of which could be exacerbated in diffusion limited environments [66]. Similarly, arginine is a metabolic connector between iron capture and reduction in oxidative stress. Arginine biosynthesis functions as a homeostasis device responsible for maintaining the equilibrium between iron uptake and oxidative-stress responses. Arginine is a precursor of polyamines, which enhance the release of pyoverdine, a sidephore that alleviates oxidative stress. They not only show high affinity for iron but also exhibit a protective role against hydrogen peroxide, thereby reducing the production of reactive oxygen species. Thus, arginine helps regulate cellular response to oxidative stress conditions [67,68], with increased arginine levels providing greater stress tolerance [68]. Arginine depletion is known to induce oxidative stress, sometimes to even lethal levels, in many bacteria [68,69,70]

Further evidence of oxidative stress can be seen in regulation of methionine and cysteine metabolism. In *Deinococcus radiodurans*, methionine and cysteine synthesis were reported to be overexpressed under simulated space-related conditions [71]. These sulfur-containing amino acids contribute significantly to the antioxidant defense system of microorganisms, and are considered to be a part of one of the most obvious microbial responses to oxidative damage. Cysteine-mediated redox signaling is an important biochemical response against oxidative damage [72], while methionines located on the surface of protein structures act as effective endogenous antioxidants to defend functionally essential molecules against oxidative damage [73]. These results suggest that oxidative stress is a major challenge to bacteria growing in space vehicles and that altering amino acid metabolism is a significant mechanism by which these organisms mitigate that stress.

One of the most important and well-characterized physiological responses of bacteria to spaceflight is an increase in biofilm formation (reviewed in [65,74,75]). Microgravity has been found to consistently increase biofilm formation in spaceflight grown bacteria, including opportunistic pathogens like *Staphylcoccus aureus* [76], *Escherichia coli* [77] and *Pseudomonas aeruginosa* [78]. Strangely, amino acid metabolism may impact this well-known phenomenon as well. A study on *K. pneumoniae* grown aboard the Shenzhou 10 spacecraft reported increased bacterial adherence capabilities thereby leading to increased biofilm formation and survival under stress conditions. Many of the genes that were upregulated included those involved in amino acid transport, metabolism and the TCA cycle [79]. Although the exact connection between biofilm formation and amino acid metabolism in spaceflight is not clear, it is generally known that increasing arginine concentrations promote biosynthesis of the second messenger c-di-GMP, which in turn is a critical regulator of biofilm formation in many bacteria. High c-di-GMP levels is suggested to promote biofilm formation. In this case, arginine appears to function both as a metabolic signal and as an environmental signal where arginine can increase c-di-GMP levels and promote biofilm formation regardless of the presence of other carbon and nitrogen sources [80,81,82]. Thus, the increased biofilm formation in *K. pneumoniae* might be in part due to enhanced flux through the TCA cycle which provides precursors for arginine. In this case, arginine has a doubly important role during spaceflight; as both a regulator of biofilm formation and as a mechanism of preventing oxidative stress. Additionally, dense biofilm communities frequently lead to the development of steep nutrient gradients, even when the environment is nutrient rich. The suggested regulatory changes in amino acid metabolism and transport, along with increased expression of TCA cycle genes, could have been to enhance the usage of amino acids as carbon and nitrogen sources [79].

Although there is a trend of increased growth rate discussed above, there are some organisms that display reduced growth. It has been proposed that one of the main reasons for an organism to reduce growth in spaceflight is radiation-induced oxidative damage to membrane lipids [13]. Changes in the membrane lipid composition under stressful environmental conditions is considered an adaptive strategy of bacteria. Several studies have reported changes tolipid metabolism in response to microgravity. For instance, in *Vibrio fischeri* simulated microgravity conditions significantly increased the shedding of lipopolysachharide during exponential growth as compared to ground control. The increased release was suggested to be associated with increased outer membrane vesicles production and the size of released particles was comparatively larger than ground controls. Later, in order to understand if the increased shedding amount of lipopolysaccharide has any impact on the integrity of the gram-negative outer membrane or cell membrane, different cell membrane-affecting agents including polymyxin B, anionic detergent and non-ionic detergent were added. The results indicated that treated cells exposed to simulated microgravity exhibited more susceptibility to disruption as compared to ground controls at all the time points tested [83]. These results suggest a change in lipid metabolism, perhaps through composition or flux. In *K. pneumoniae* strains after growth on the Shenzhou 10 spacecraft, the genes involved in fatty acid metabolism were suggested to be downregulated [79]. Likewise, *B. subtilis* spores and *Rhodospirillum rubrum* exposed to International Space Station and modeled microgravity respectively showed down-regulation of genes encoding lipid biosynthetic enzymes [84,85]. The metabolic pathways associated with fatty acid metabolism, phospholipid biosynthetic process, and cellular lipid biosynthetic processes in *E. coli* and *Enterococcus faecium* strains after spaceflight were also affected [86,87]. However, it is not clear if the downregulation of lipid metabolism is a result of oxidative stress. The downregulation of lipid biosynthesis could be due to reduced need for cell membrane synthesis in non-growing cells [84].

### 3.2. Secondary Metabolism

Secondary metabolites (also referred to as specialized metabolites), are bioactive compounds that can have potential advantageous properties including antioxidant, growth-promoting, anti-allergic, anti-inflammatory, anticancer, antihypertensive and antimicrobial properties, or can have potentially negative properties such as being carcinogenic or toxic/pathogenic [88,89] Secondary metabolites have also been reported to help maintain cellular homeostasis by regulating carbon and nitrogen flow in the cell [90], by re-generating intracellular NAD^+^ concentrations [90,91,92] and by relieving cellular oxidative stress [93]. Most of these molecules are polyketides and non-ribosomal peptides and are produced by biochemical pathways that are encoded in discrete gene clusters. Some of these gene clusters synthesizing secondary metabolites are either silent or expressed at very low concentrations under normal terrestrial conditions. However, stressful conditions can lead to activation of such gene clusters [94,95,96]. This means that the unusual combination of stresses experienced by bacteria during spaceflight has the potential to induce cryptic gene clusters, thereby changing the chemical diversity of metabolites in new and unpredictable ways Understanding the influence of microgravity on the production of secondary metabolites by bacteria is worthwhile to develop strategies to mitigate the harmful impacts on life science or infrastructure aboard space-faring vehicles.

Thus far, studies on secondary metabolism have focused on only a few bacteria (mainly *Streptomycetes*, *Escherichia coli*, and *Bacillus*) and are mostly limited to one or a few metabolites per study (Table 1). These studies have suggested altered secondary metabolite production levels, but the specific responses have been unique to each species. One study used Ground-Based Facilities to assess the impact of simulated microgravity on three types of secondary metabolites. These included the peptide antibiotic Gramicidin S by the unicellular aerobic bacterium *Bacillus brevis* [97], β-lactam antibiotics by the filamentous aerobic bacterium *Streptomyces clavuligerus* [98] and rapamycin by the filamentous aerobic bacterium *Streptomyces hygroscopicus* [99]. Both the β-lactam antibiotics and rapamycin were produced at lower levels than those observed under normal gravity in the same type of rotating wall bioreactor, with a maximum 90% decrease in rapamycin, whereas Gramicidin was unaffected [100]. In a different study, Nikkomycins-producing strains of *Streptomyces ansochromogenus* were investigated to understand the biological response to production onboard a satellite for 15 days. The production of Nikkomycins in nearly all strains was reported to be increased by 13-18%, with increases specifically in Nikkomycin X and Z [7,101]. Nikkomycins are nucleoside peptide antifungal agents that function by inhibiting fungal chitin synthetases and cause osmotic lysis. Because of this property they can have significant application on agricultural practices and may be useful in spaceflight grown crops [102].

In a paired spaceflight and ground control-based study of *Streptomyces plicatus*, samples were grown for 12 days in defined and complex media. A volume from both media samples was fixed at 7 and 12 days to test for antibiotic production, and the residual volume was maintained at 6°C for the rest of the 17 day mission. In ground samples, production of the antibiotic actinomycin D reached a maximum on day 7 (the first sampling day) in both media types (defined media: 0.96 µg/mg, complex media: 0.47 µg/mg), and productivity dropped with further incubation. On the other hand, the productivity of the spaceflight sample grown in defined medium continued to increase with culture age throughout the 17-day mission reaching a maximum of 0.93 µg/mg on day 17, while in complex medium the productivity of the space samples reached a maximum (1.02 µg/mg) on day 12 (Table 1) with a slight drop in productivity on day 17 [7,103]. This meant that in defined medium, the maximum yield for both space-grown and ground samples during the 17-day mission was the same but the space-grown samples took much longer to get there. Similarly, in complex media, space –grown samples reached maximum productivity later than ground controls but the maximum yield was 115% higher than that for ground controls. These results suggested that the production time course was delayed for space-grown samples compared to ground controls, but since productivity was still on the rise for space-grown samples in defined media, additional duration or long-term experiments were needed to fully evaluate the process kinetics [7,103]. With the goal to understand if longer exposure to weightlessness would enhance the productivity of actinomycin D, another experiment on *Streptomyces plicatus* was conducted for 72 days onboard the International Space Station. This study showed that as compared to ground controls, the actinomycin D concentration in space was 15.6% higher on day 8 and 28.5% higher on day 12 while samples taken on day 16 and beyond showed lower production as compared to ground controls [104]. These results corroborated the previous finding of increased actinomycin production at around day 12 of spaceflight samples, but the longer duration of the second study revealed a total negative impact on yield of actinomycin D in space. Though the specific causes and mechanisms responsible for the initial stimulation of productivity in spaceflight are unknown, in the first study it was suggested that the slow growth rate of the space flight cultures may have resulted in higher specific productivity yields than faster-growing ground control cultures. An additional complication is that the latter study used an active feed of production medium to maintain viable cultures for the 72-day mission while the former study didn’t actively feed media over the duration of mission. Regardless, these studies show a remarkably different profile of secondary metabolite production in spaceflight compared to terrestrial growth. Similarly, a study on *Cupriavidus metallidurans* under simulated microgravity showed increased production of the polyester polymer poly-b-hydroxybutyrate after 24 h, but not after 48 h, compared with ground controls (Table 1) [105]. Poly-b-hydroxybutyrate is a common bacterial carbon storage polymer which is formed when carbon–nitrogen ratio is high [106]. This result is interesting given the dynamics of carbon utilization discussed above, as it suggests that this organism is not initially starved for carbon, but becomes so after approximately 24 h. This result also provides an example of the interplay between primary and secondary metabolism.A study on cyanobacterium *Microcystis aeruginosa* reported a higher level of the toxin microcystin under simulated microgravity condition as compared to control on days 2 to 6. Additionally, twice as much microcystin was found in the extracellular medium of the simulated microgravity sample as the control after 2 days of incubation (Table 1). Increased extracellular microcystin concentration under terrestrial conditions has usually been assumed to be the product of cell lysis and peptide leakage. However, in this study the increased extracellular microcystin concentration was probably due to active microcystin release since the researchers did not see an increased number of deformed or dead cells under direct microscopic observation. Enhanced microcystin production was reported to be associated with the increased photosynthetic pigment concentration under simulated microgravity as compared to control, which was suggested to be due to their increased light harvesting function. Despite the same doses of light provided under both simulated microgravity and ground control, it appeared that cells treated with simulated microgravity needed to harvest more light to be used in photosynthesis and meet the high energy consumption for microcystin synthesis as compared to gravity control. Nitrogen is a key element for microcystin synthesis, therefore it was unsurprising that significantly higher nitrogen absorption under simulated microgravity was observed on day 4 and 6 than that of control. The results indicate that enhanced microcystin synthesis under simulated microgravity could make *M. aeruginosa* more dangerous [107]. Cyanobacterial blooms are dangerous events on Earth, but in the real weightless space environment, where there is limitation in the flow of liquid with accumulated byproducts along with various stresses, cyanobacterial blooms may occur more often or be even more toxic. This may cause serious health and ecosystem risks aboard space-faring vehicles. Also, the symbiotic association of plant and cyanobacteria, including *M. aeruginosa* [108], can ultimately compromise the safety of astronauts’ health via the food chain. The occurrence of cyanobacterial mass populations can create a significant water quality problem by synthesizing a wide range of odors, noxious compounds, or potent toxins. Thus, besides direct and indirect intake, the stronger microcystin release induced by microgravity may impact the recycled water system, risking the biological system, including plants, in a closed controlled spaceflight environment [107].

A study on *S. coelicolorA3* was conducted under both simulated microgravity and in Shenzhou-8 spaceflight along with static controls on ground, and simulated 1-g control in spaceflight. The major objective was to understand the effect of spaceflight on the bacteriostatic activity of *S. coelicolorA3* against *B. subtilis* when the bacteria were cultured together. The results indicated that *S. coelicolor* exhibited stronger bacteriostatic activity against *B. subtilis* under microgravity as compared to gravity control. Interestingly, the production of actinorhodin and undecylprodigiosins, two well-known antibiotics produced by *S. coelicolor*, were reported to be reduced and unaffected respectively (Table 1). These results were reported under both real and simulated microgravity, and were supported by transcriptomics data. This implies that there were other bioactive substances or secondary metabolites produced under microgravity enhancing the bacteriostatic activity. In addition to actinorhodin and undecylprodigiosins, *S. coelicolor* also produces a calcium-dependent ionophore antibiotic (CDA), methylenomycin (MMY), and a cryptic polyketide (CPK). Of the three, the cryptic polyketide was upregulated, suggesting that it was responsible for the bacteriostatic properties under microgravity, though another as-yet-uncharacterized metabolite cannot be ruled out [109].

The lack of shear stress in microgravity has not only been suggested to impact secondary metabolite production but also has been demonstrated to impact secondary metabolism accumulation sites. For instance, while production of the peptide antibiotic microcin B17 by *Escherichia coli ZK650* was inhibited by low-shear simulated microgravity, the accumulation site of microcin was found to be markedly different depending on whether *Escherichia coli* was grown in shaking flasks or rotating wall bioreactors (RWBs). When cells were grown in flasks in normal gravity, the majority of the microcin was associated with the cells. However, when the cells were grown in RWBs, the microcin accumulated in the extracellular medium (Table 1) [110]. This location dependence was confirmed to be the result of the lack of shear stress by the addition of a single teflon bead (1/8 in. diameter) to the medium. In normal gravity mode the beads do not move freely and remain at the periphery of the reactor, while in simulated microgravity the beads move freely throughout the liquid and impose high degree of shear stress in the bioreactors [111]. Addition of even a single bead changed the site of accumulation from 91% extracellular to 98% cellular [111], while adding beads in the normal gravity mode had only a partial effect. The results from *Escherichia coli* ZK650 were specifically analyzed in further experiments to understand if the low shear environment impacts the production of secondary molecules. A single Teflon bead added to the medium in the simulated microgravity mode was compared with the rotating wall bioreactor without the bead and with shaken flasks. The results suggest that the growth was stimulated slightly and microcin B17 production was considerably increased in the RWB with added teflon bead (Table 1). This response is remarkably specific. The addition of 25 beads to cultures of *Streptomyces hygroscopicus* in RWBs under simulated microgravity did not affect the distribution of rapamycin. Simulated microgravity inhibited rapamycin production and favored extracellular accumulation with and without addition of beads [99].

All these results suggest that effects of microgravity on secondary metabolism may be specific depending on the strain, growth condition, pathway utilized, or time course analyzed (Table 1). Furthermore, past studies are either limited to already known metabolites or have focused on bacteria which are already well-known metabolite producers. However, space vehicles hold diverse species whose behaviors are unstudied and could have responses under microgravity beyond prediction. Additionally, understanding microbes at a global metabolomics level could provide more comprehensive knowledge about the overall responses exhibited under microgravity. For example, even though the levels of secondary metabolites actinorhodin and undecylprodigiosins had reduced expression and metabolic level, *S. coelicolor* still had increased bacteriostatic activity [109], possibly due to induction of other secondary metabolites. Utilizing global metabolomics approaches may provide a more comprehensive understanding of the impact of microgravity on secondary metabolism.

**Table 1:** Secondary Metabolites Production to Simulated Microgravity and Spaceflight.

Organism	Metabolite	Impact of Microgravity	Experimental Location	References
*Bacillus brevis* strain Nagano (1997)	Gramicidin S	Unchanged production level	Simulated microgravity (Single High-aspect rotating vessels (sHARV))	[97]
*Escherichia coli* ZK650 (1997)	Microcin B17	Decreased production with extracellular accumulation	Simulated microgravity (High-aspect rotating vessels (HARV))	[110]
*Escherichia coli* ZK650 (2001)	Microcin B17	Increased production with shear stress (teflon bead)	Simulated microgravity (Rotating-wall bioreactors (RWV))	[111]
*Streptpomyces clavuligerus* NRRL 3585 (ATCC 27064) (1997)	β-lactam antibiotics	Decreased production	Simulated microgravity (Single High-aspect rotating vessels (sHARV))	[98]
*Streptomyces ansochromogenus* 7100 (1998)	Nikkomycin, Nikkomycin X, Z	Increased by 13–18 %	Space flight (15 days)	[101]
*Streptomyces hygroscopicus* ATCC 29253 (2000)	Rapamycin	Decreased production with extracellular accumulation site	Simulated microgravity (Rotating-wall bioreactor (RWB))	[99]
*Streptomyces plicatus* WC56452 (2002)	Actinomycin D	Increased production with altered time course	US Space Shuttle mission STS-80	[103]
*Streptomyces plicatus* WC56452 (2006)	Actinomycin D	Increased concentration at day 8 and 12 with decrease after	International space station (ISS)	[104]
*Streptomyces coelicolor* A3(2) (2015)	Undecylprodigiosin (RED)	Unchanged production amount, earlier production time	2D-clinostat	[109]
*Streptomyces coelicolor* A3(2) (2015)	Actinorhodin (ACT)	Decreased production	2D-clinostat	[109]
*Streptomyces coelicolor* A3(2) (2015)	Undecylprodigiosin (RED)	Decreased production	Shenzhou-8 Space mission	[109]
*Streptomyces coelicolor* A3(2) (2015)	Actinorhodin (ACT)	Decreased production	Shenzhou-8 Space mission	[109]
*Cupriavidus metallidurans* LMG 1195 (2009)	Poly-β-hydroxybutyrate (PHB)	Increased production at 24 h and decrease after 48 h	Simulated microgravity (Rotating wall vessel (RWV))	[105]
*Microcystis aeruginosa* PCC7806 (2010)	Microcystin	Increased production with extracellular accumulation	Simulated microgravity (Rotary cell culture system (RCCS))	[107]

### 3.3. Link between Primary and Secondary Metabolites

At the terrestrial level, the intimate link between primary metabolic pathways and many secondary metabolic pathways has been demonstrated [112]. It is well understood that some enzymes of primary metabolism catalyze the formation of products that can be channeled into the pathways of secondary metabolites [113]. Glucose degradation via the pentose phosphate cycle forms erythrose-4-phosphate, which can react with phosphoenolpyruvate to yield shikimic acid, an intermediate product connecting primary and secondary metabolism [114]. Shikimic acid is a precursor for many aromatic amino acids including tryptophan and tryptophan derivatives. There are many tryptophan-derived secondary metabolites, including serotonin and indole alkaloids [115]. Similarly, 3-phosphoglyceraldehyde generated via glycolysis is converted to pyruvate, and subsequently to acetyl-coenzyme A, the most predominant building block of secondary metabolism. The condensation of three acetyl-CoA units gives rise to mevalonic acid [116] a key intermediate in terpene biosynthesis. Additionally, acetyl-CoA can condense with oxaloacetate as part of the tricarboxylic acid (TCA) cycle, providing a source for carbon skeletons for several amino acids [116,117,118] which are common starter units for secondary metabolites [118]. It is reported that polyketide biosynthetic clusters recruit the malonyl-CoA:ACP transacylase enzyme, a starting unit from the fatty acid biosynthetic machinery [112,119]. This establishes a balance between fatty acid and polyketide biosynthesis that effectively sets the upper limit on polyketide yields, manifested at the level of precursor supply, malonyl-CoA:ACP transacylase availability, or both. The reported changes in gene expression of enzymes involved in glycolysis, TCA cycle, and amino acid metabolism under microgravity clearly shows that primary metabolism is often globally impacted. This in turn will impact secondary metabolite production, which has been seen in several studies. However, none of the published studies so far have investigated or addressed the connections between primary and secondary metabolism under spaceflight conditions. To better understand the bacterial metabolic response to spaceflight not only do primary and secondary metabolism need to be investigated further, but the dynamic interplay between them needs to be analyzed as well.

## 4. Concluding Remarks and Future Directions

In space, bacterial physiology changes on a global scale due to the stresses imposed by the environment around the cell [13]. Metabolic changes affect the diverse biological activities of microorganisms under microgravity. Studies so far have mostly used transcriptomics and proteomics, while the cellular metabolome is largely untouched. Where transcriptomics and proteomics integrate the linear predictive power of the genome, the metabolome represents the nonlinear, final biochemical products of the genome. Understanding at a systems level can be enhanced by integration of metabolomics with additional omics levels datasets. Hopefully a more comprehensive systems-level understanding will increase the predictability of microbial responses to microgravity.

Moreover, the collective understanding of transcriptomics and metabolomics associated in formation of a specific metabolite would be invaluable for researches that wish to engineer, isolate, or sequence the genes of interesting biosynthetic clusters. Bacteria offer a wealth of potential for the discovery of new and important microbial products. Microbial products have obvious utility in medicine and biotechnology, but they are also important for their effects on microbial communities in other biological systems, such as plants. Broadening the horizon of bacterial species and understanding the altered levels under microgravity could offer unique advantages not only for bioprocessing industries but also enhance plant growth aboard space vehicles. The use of engineered microorganisms to produce primary or secondary metabolites is becoming more common in bioprocessing technology and a large number of chemicals, pharmaceuticals, biofuels and agricultural compounds have been produced at high enough efficiencies through metabolically engineered microorganisms [120]. It is suggested that modern bioprocessing technology is highly dependent upon chemical and physical environmental parameters. There remains much to discover about the nature of diverse secondary metabolisms in such stressful environments of spaceflight. The changes of environmental factors such as temperature, oxygen availability, and diffusion limitations under microgravity can provide a condition which can be harnessed in a best way possible to be used for engineered microorganisms to generate the useful metabolites. Therefore, understanding the specific cause-and-effect mechanisms of microbial responses to microgravity at the molecular level could provide ground breaking discoveries for not only space applications and other biotechnological industries, but also could be advantageous for future human spaceflight missions. 

## Data Availability

Not applicable.
